# Density Functional Theory-Fed Phase Field Model for Semiconductor Nanostructures: The Case of Self-Induced Core–Shell InAlN Nanorods

**DOI:** 10.1021/acs.cgd.4c00316

**Published:** 2024-05-14

**Authors:** Manoel Alves Machado Filho, William Farmer, Ching-Lien Hsiao, Renato Batista dos Santos, Lars Hultman, Jens Birch, Kumar Ankit, Gueorgui Kostov Gueorguiev

**Affiliations:** † Thin Film Physics Division, Department of Physics, Chemistry and Biology (IFM), Linköping University, Linköping SE 581 83, Sweden; ‡ Machadornos LTDA - Cursos, Mentoria e Consultoria, Rua Lindolfo Rocha, 47−2° Centro, Jequié, Bahia 45200-120, Brazil; § Materials Science and Engineering, School for Engineering of Matter, Transport and Energy, Arizona State University, 551 E. Tyler Mall, Tempe, Arizona 85287, United States; ∥ Instituto Federal de Educação, Ciência e Tecnologia Baiano, Itaberaba, Bahia 46880-000, Brazil

## Abstract

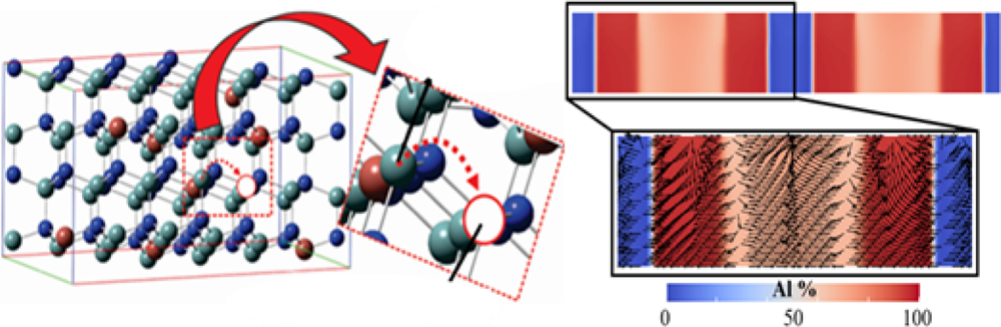

The self-induced formation of core–shell InAlN nanorods (NRs) is addressed at the mesoscopic scale by density functional theory (DFT)-resulting parameters to develop phase field modeling (PFM). Accounting for the structural, bonding, and electronic features of immiscible semiconductor systems at the nanometer scale, we advance DFT-based procedures for computation of the parameters necessary for PFM simulation runs, namely, interfacial energies and diffusion coefficients. The developed DFT procedures conform to experimental self-induced InAlN NRs’ concerning phase-separation, core/shell interface, morphology, and composition. Finally, we infer the prospects for the transferability of the coupled DFT-PFM simulation approach to a wider range of nanostructured semiconductor materials.

## Abstract 2

The formation of core−shell InAlN nanorods (NRs) is addressed at the mesoscopic scale by DFT-resulting parameters to develop a phase field modeling (PFM). Left panel: DFT procedure for determination of the diffusion coefficients for N, In, and Al atoms for typical cores and shells of the InAlN NRs. Right panel: 2D evolution of an InAlN NR simulated by PFM.

## 1 Introduction

The phase-field model (PFM) is a well-known technique to simulate nanostructure evolution at diffusive time scales. It has been extensively applied to analyze microstructural evolution during solidification and solid-state phase transformations.^1−3^ The phase-field model’s popularity in investigating material processes is due to that it treats moving interface problems without the necessity to explicitly track these interfaces. The inherent diffuse interface technique allows for facile coupling of thermodynamic driving forces, such as elastic misfit energy and interfacial and chemical free energies, with atomic transport processes, such as diffusion and advection. Self-organization in phase-separating binary and ternary metallic alloy films has been extensively investigated using the PFMs, wherein several known variants with lateral (LCM), vertical concentration modulations (VCM), and also random concentration modulations (RCM) were simulated and compared to transmission electron microscopy (TEM)-characterized films synthesized by physical vapor deposition (PVD).^4^ These models have also been employed to predict the processing conditions under which novel hierarchical variants could be synthesized in binary^5,6^ and ternary^7^ alloy films. More recent extensions of the PFM are able to simulate the formations of hillocks and narrow channels on a film’s surface.^8,9^

So far, the PFM has been employed to analyze three different types of interfaces: (i) the free surfaces of a crystal (solid/vapor interface like in refs (8,9)), (ii) grain boundaries, and (iii) interphase interfaces (alpha/beta interfaces, etc.). For the latter, PFM has been successfully applied to study the formation mechanism of the core–shell (C–S) structures obtained by sintering of spherical nanoparticles.^10^

While the PFM has been already conceptually developed with the nanostructural evolution in PVD-grown phase-separating ternary alloy films,^7^ it has not yet been applied to simulate the evolution of experimentally demonstrated self-induced semiconductor nanostructures. This aspect, together with the idea to feed the PFM by parameters obtained via sophisticated DFT calculations, is the motivation for our present work on demonstrating the capabilities of PFM simulations for understanding the formation of group IIIA nitride ternary nanostructures. The PFM model introduced here is deliberately reduced to 2D in order to investigate its basic reproducing capabilities and features. The present development model can be seen as a precursor and a prerequisite for a full 3D PFM model of semiconductor nanostructures. The chosen demonstrator case, namely, the self-induced core–shell InAlN nanorods (NRs), belongs to a class of materials attracting strong research interest. Group IIIA nitrides consisting of AlN, GaN, InN, and especially their ternary and quaternary alloys reunite valuable electronic and engineering properties, including a bandgap that is tunable in a wide range from near-infrared (InN ∼ 0.64 eV) to deep ultraviolet (AlN ∼ 6.2 eV). Out of the three crystal structures wurtzite (wz), zinc-blende, and rock-salt, the wz-structure, consisting in two interpenetrating hexagonal sublattices composed of the group IIIA atoms and the nitrogen atoms, respectively, combines high thermal stability with high chemical inertness, and even high break down voltage.^11,12^ These valuable properties enhance the prospects of group III nitride materials for applications extending to LEDs with high brightness, solar cells, etc.

The wz-structure is observed in wide variety of group IIIA nitride systems–from bulk crystals of AlN and GaN grown for example by physical vapor transport,^13^ to thin films of group III nitrides grown by metal organic chemical vapor deposition (MOCVD),^14^ molecular beam epitaxy (MBE), and magnetron sputter epitaxy (MSE)^15^ on various substrates including sapphire, SiC, and Si. In recent years, one of the rapidly maturing lines of research in the field of group IIIA nitrides focuses on variety of nanostructures such as self-induced core–shell nanorods (NRs).^16−18^

Growth of group IIIA nitride NRs has been approached by a diversity of methods,^19−21^ which is also due to their simplified fabrication not requiring catalysts or any advanced lithography and substrate pretreatments.^22,23^ Part of our team has reported on the core–shell formation in self-induced InAlN NRs by growing them directly onto electron-transparent amorphous carbon (a-C) substrates using reactive magnetron sputter epitaxy (MSE).^18^ Scanning transmission electron microscopy (S)TEM analysis was employed to address the structural and compositional evolution of the core–shell InAlN NRs in their stages of nucleation of discrete In-enriched and Al-rich islands that grow and coalesce during the NR axial-radial growth. The distinctive core–shell structure is attributable to separate nucleation of Al-rich and In-enriched islands due to formation of interfaces with diverging interfacial energies and chemical potentials. The In-enriched domains feature a higher growth rate, thus forming the core, while the shell results from the slower growing surrounding Al-rich shell.^18^ Formation of self-induced NRs is by far not limited to group III nitrides, but a much broader phenomenon observed throughout a wide variety of ternary alloys, some of the examples being InGaAs, InGaP, and ZnMgO.

Modeling of NRs growth evolution based on the density functional theory (DFT) methodology can provide quantitative insights into their structural and electronic properties. In a previous work, we investigated precursor prevalence and energetics employing the DFT-based synthetic growth concept (SGC) to elucidate the formation mechanism of the self-induced InAlN core–shell nanorods (NRs) synthesized by reactive magnetron sputter epitaxy (MSE).^24^ Yet, interfacial phenomena, diffusion, and LCM, VCM, are difficult to account for by applying DFT or SGC only. Suitable mesoscopic modeling methods such as PFM emerge as suitable methods of choice in order to achieve further insight into such features and growth phenomena.

In this work, we present steps building a DFT-fed PFM model for simulating a semiconductor nanostructured material system: from employing specially designed DFT calculations and developing computing schemes for obtaining the parameters necessary for running PFM simulations. After using DFT to compute the interfacial energies as well as the atomic diffusion coefficients for In, Al, and N atoms, we incorporated these parameters into the PFM model of the InAlN core–shell NRs. The PFM results not only corroborate experimental findings on the higher In content in the NRs cores and higher Al content in the NRs shells but also provide the driving forces behind such compositions, e.g., behind the diffusion processes of In atoms across the interface from NRs poorer to these atoms regions to In richer regions.

## 2 Methodology and Computational Details

### 2.1 DFT Calculations

Except where explicitly stated, the calculation setup adopted in this work is the one of the density-functional theory (DFT) within its Generalized Gradient Approximation (GGA) at the Perdew–Burke–Ernzerhof (PBE) level of theory as implemented in the Quantum Espresso code,^25,26^ which employs plane-wave basis sets and projector augmented wave method (PAW) pseudopotentials. The methodology chosen for this work takes into account a recent comparative study of different levels of theory and their performance in terms of accuracy and efficiency in the specific context of the core–shell InAlN NRs.^27^

Test calculations for comparative purposes of the interfacial energy values have been carried out using the Gaussian code^28^ and the PBE as well as the PW91 levels of theory.

For the purposes of obtaining the interfacial energies and the diffusion coefficients at the InAlN interfaces, the kinetic energy and the charge cut offs are chosen as 100.0 and 500.0 Ry, respectively. For the structural relaxations of the model systems representing core–shell InAlN NRs, their atomic constituents were allowed to relax until the forces decrease to values less than 0.01 eV Å^–1^. The energy convergence criterion in geometry optimizations was 10^–5^ eV. In order to avoid spurious interactions with images of supercells of the NR, a sufficiently large vacuum ring (25 Å) around the NRs structures was employed in all relevant calculations.

InAlN model systems with varying elemental compositions were first relaxed and then employed for the purposes of calculation of the interfacial energies and the diffusion coefficients, whereby the periodic boundary conditions (PBC) were customized to assemble models with different dimensionality/finiteness, namely, infinite as well as semi-infinite, i.e., featuring a free surface. In order to ensure that the results obtained for the interfacial energies and diffusion coefficients do not depend on the supercell sizes, we tested the impact of different NRs supercell sizes on the results. Such tests are particularly indispensable for an accurate simulation of vacancy creation and the corresponding hopping events, as adopted in this work for the calculation of diffusion coefficients.

The determination of the diffusion coefficients requires calculation of energy barriers overcome by the hopping atoms that are driving the diffusion processes. For estimation of these energy barriers, the nudged elastic band (NEB) method was employed.^29^ NEB represents an adequate, well-developed, and powerful numerical tool to determine the minimum energy paths (MEPs) and the transition states between known reactants and products or in a more general context when chemical bonds are broken and new ones subsequently formed. For this purpose, NEB relies on the estimation of a sequence of discrete “images” of the model system, which may include, when applicable, the surroundings of the directly reacting species, which is the case when energy barriers are determined for diffusing/hopping species within a solid-state model system, e.g., the self-induced core–shell NRs. Each “image” corresponds to a specific geometrical layout of the atoms on their way from a fixed initial to a fixed final state. As initially postulated, standard NEB calculations are performed for approximately 100 ionic steps to achieve an approximate convergence for a specific MEP.^29^ In order to obtain a rigorous convergence to a transition state associated with a specific MEP, the standard NEB calculations are “upgraded” by applying the so-called climbing-image NEB algorithm (CI-NEB),^30^ thus ensuring that the “image” reaches the exact saddle point. In all energy barrier calculations performed in this work for the purposes of the self-induced core–shell NRs, the number of “images”(including the initial and final “images”) is set to eight, which is important because of the correlation between a higher number of “images” employed in a NEB calculation and an improved precision of a MEP determination. The NEB code is applied successfully to solving a wide range of problems, including growth of group III nitride materials^31,32^ with some of the most recent examples dedicated exactly to determination of energy barriers in relation to investigation of diffusion processes.^33,34^

### 2.2 Theory of the Phase Field Model and Its Setup for the Simulation of the NRs

In this section, we describe a phase field model that utilizes a coupled system of Cahn–Hillard and Allen–Cahn models to simulate nanorod growth. The evolution of the nanorod is governed by the minimization of the total free energy expressed as functional (eq 1B). While the phase-field order parameters, ϕ_Al_ and ϕ_In_, correspond to the concentration of Al and In phases respectively, the concentration of N is represented by ϕ_N_. Therefore, the conserved order parameters obey
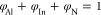
1

The nonconserved order parameter, η_1_, η_2_, and η_3_, attain the maximum value of 1.0 within the Al-rich, In-rich, and vapor phases, respectively, as they smoothly transition to 1.0 across the phase boundaries. Consequently, η_1_ and η_2_ attain a minimum value of 0.0 in the vacuum phase surrounding the nanorod. The free energy functional, *F*, can be expressed as
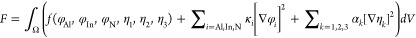
2where the first two terms represent the chemical free energy of the phases, *κ*_*i*_ are the gradient energy coefficients for the corresponding concentration fields of Al, In, and N, and the prefactors, α_k_, represent the phase gradient energy coefficient. The chemical free energy is given by
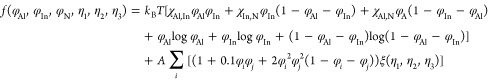
3where *A* is an energy term to scale the interaction between the concentration fields and the interface of the phases and
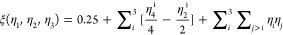
4

Based on the variational principles, the concentration evolution equations can be written as
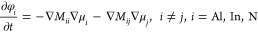
5where μ_*i*_ is the chemical potential of component *i* and is expanded below from the variational derivative of the free energy functional with respect to concentration as
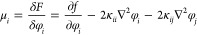
6

Here, the gradient energy coefficients follow the order κ_AlIn_ = κ_Al_ + κ_In_, κ_InIn_ = κ_In_ + κ_N_, and κ_AlIn_ = κ_InAl_ = κ_N_. The Onsager kinetic coefficients, *M*_*ii*_ and *M*_*ij*_ (i ≠ *j*, i = Al, In, N), are defined as
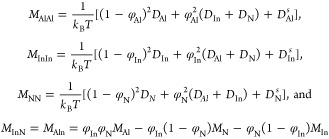
7where, *T* is temperature, *D*_*Al*_, *D*_*In*_, and *D*_*N*_ represent the bulk substitutional diffusion coefficients of respective elements while the superscript “*S*” indicates corresponding surface diffusion coefficients. Interfacial energies and diffusion coefficients are derived from the DFT-based simulation procedures described in the next section. Equation 7 is very similar to a Nernst–Einstein (NE) relationship, which is typically used to correlate atomic mobility with diffusion coefficients. The additional surface diffusion term accounts for the enhanced mobility of atoms along the surfaces. It is to be noted that the NE relationship is independent of spatial dimensions; i.e., it is equally applicable in 2D as in 3D.

The coupled nonconserved order parameters are evolved using
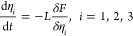
8

All the constants are nondimensionalized using the characteristic length (l* = (κ_*i*_/2*k*_*B*_*T*)^1/2^Δ*x*) and the time (t* = (*k*_B_*T*/*M*_ii_^*^l^*2^)), where *M*_ii_^*^ is the dimensional atomic mobility and *k*_B_ is Boltzmann’s constant, as defined in our previous work.^4,5,7^ Finally, we apply the method of finite differences to solve the above system of partial differential equations at spatial and temporal resolutions of Δ*x* and Δ*t*, respectively. Periodic boundary conditions are applied along the left and right edges of the computational domain. Further, imposing zero flux of the order parameter along the top and the bottom edges ensures 90° contact angles along the top and the bottom edges of the domain. These contact angles essentially result from the equilibrium of surface tensions at the junction of the two-phase interfaces and free surfaces. To account for this equilibrium in phase-field simulations, a suitable Neumann-type boundary condition, as described elsewhere,^35^ can be used.

**Figure 1:**
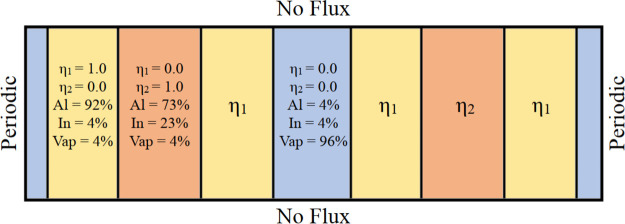
Schematic diagram showing the initial condition. The yellow, orange, and blue phases represent the Al-rich (η_1_), In-rich (η_2_), and N-rich (η_3_) phases, respectively. The percentages as listed in the figure sum up to the metallic species, i.e., 50% of all atomic species, with the remaining 50% being implicitly reserved for the nitrogen species. For numerical calculations, no flux boundary conditions (BC) are imposed along the top and the bottom edges of the computational domain, while periodic BCs are assumed along the left and the right.

The initial condition comprises two nanorod seeds, each of width 25Δ*x* with an Al-rich phase surrounding the In-rich phase as shown schematically in Figure 1. Corresponding concentrations of each phase are also specified.

A minimum of 4% of the minority component had to be maintained to ensure the numerical stability of the model. The nondimensional deposition rate is defined as (ν = 1/*n*Δ*t*), where Δ*t* is the dimensionless time step for numerical integration and *n* is the number of time steps between the deposition of consecutive layers. To simulate deposition, an additional layer of noisy concentration field (which preserves the overall composition of elements in the domain) is deposited on the nanorods every 15Δ*t*. We would like to clarify that the chosen value of deposition rate corresponds to the simulation of phase separating nanostructures with lateral concentration modulations that has been reported in our previous work.^7^ The nanorod height is allowed to increase until 260Δ*x* as the phase separation continues.

## 3 Results and Discussion

### 3.1 Interfacial Energies

**Figure 2:**
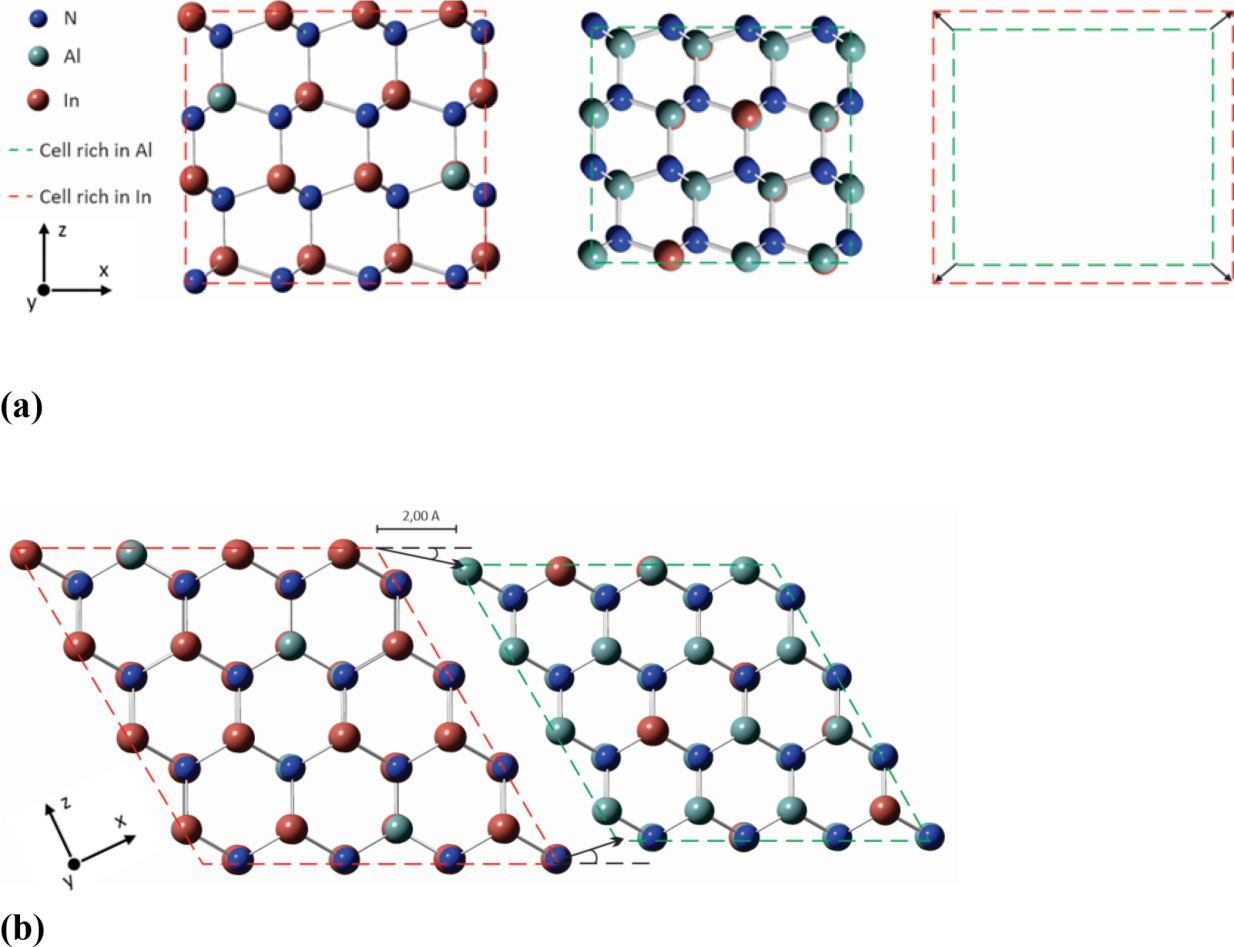
Illustration of the procedure for calculation of the interfacial energies relevant to the core–shell InAlN NRs. (a) Any combination of two InAlN wurtzite phases labeled as phase (1) and phase (2) in Table 1 and composed of In- and Al-rich cells, respectively, presented in scale to underline the lattice mismatch. (b) Bringing into contact the two InAlN wurtzite phases and forming interface between them. Here, the starting point of the relaxation process is at a distance of 2.0 Å.

As described by Demkowicz et al.,^36^ the interfacial energy can be derived from a schematic model of the interface resulting from bringing in close contact a “bilayer” of the two phases in question, which in the simplest approximation to the case of InAlN NRs are two semi-infinite bulk model systems of the AlN and InN compounds, respectively, or their slab-like analogues which are thick enough, e.g., > 6 nm, to ensure preserved wurtzite structure. Thickness is measured along the deposition axis, which is denoted as the *z* axis on all subsequent figures. This model system for calculating the interfacial energies is shown in Figure 2 a. The interfacial energy can be expressed as
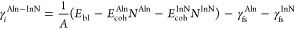
9where γ_i_^AlN-InN^ is the interfacial energy per unit (supercell) area; γ_fs_^AlN^ and γ_fs_^InN^ are the energies of the wurtzite AlN and InN free surfaces, respectively; *E*_bl_ is the total energy of what traditionally in such model systems is called “a bilayer”,^36^ and, in a more general case, is the system consisting in the two-phase brought into contact; *E*_coh_^AlN^ and *E*_coh_^InN^ are the cohesive energies per atom for perfect wurtzite AlN and InN, while N^AlN^ and N^InN^ are the number of atoms per supercell (N^AlN^ = N^InN^ = 64 in the present case); *A* is the interface surface per unit (supercell) area.

The cohesive energies, which describe, at the PBE level of theory, the AlN and InN phases (Table 1), as well as the related energies of the AlN and InN free surfaces (i.e., semi-infinite model systems, with a free surface of the same structure and area as the interface of the joint AlN-InN model system), are easily obtainable by simulating wurtzite AlN and InN systems with periodic boundary conditions (PBC).

**Table 1:** Interfacial Energies Computed According to eq 1 and Matching Figure 2 for Different Combinations of Two InAlN Phases (1) and (2), Forming an Interface in Self-Induced Core–shell NRs^a^

combinations of two InAlN phases (1) and (2), forming an interface in self-induced core–shell NRs	benchmark case: pure AlN to pure InN	Al_0.70_In_0.30_N to Al_0.99_In_0.01_N	Al_0.75_In_0.25_N to Al_0.98_In_0.02_N	Al_0.79_In_0.21_N to Al_0.89_In_0.11_N	Al_0.89_In_0.11_N to Al_0.97_In_0.03_N
cohesive energies, *E*_coh_, eV/at. for phases (1) and (2)	–7.114^(1)^	–6.511^(1)^	–6.629^(1)^	–6.523^(1)^	–6.769^(1)^
	–5.285^(2)^	–7.090^(2)^	–7.055^(2)^	–6.769^(2)^	–7.011^(2)^
γ_i_^(1)-(2)^, mJm^–2^	979.2	701.1	655.3	629.1	523.8

a Besides the interfacial energy corresponding to the hypothetical benchmark case of interface between pure AlN and InN phases, the other interfacial energy values characterize interfaces of direct relevance to as-grown or potentially synthesizable NRs

Contrastingly, to compute *E*_bl_ is more challenging due to the difficulty to geometrically relax the two-phase, interface containing structure shown in Figure 2 a. The AlN/InN lattice mismatches of 12.3% and 11.8% for the lattice constants “*a*” and “*c*”, respectively, obtained in this work after relaxation of the AlN (*a* = 3.13 Å; *c* = 5.02 Å) and InN (*a* = 3.57 Å; *c* = 5.74 Å) wurtzite compounds, are similar to the accepted in the literature AlN/InN lattice mismatch values of 11% −13%.^37^ When two substantially mismatching lattices, such as those of InN and AlN, form an interface, the relaxation of the model system joint at the interface is not straightforward. To overcome the lattice mismatch difficulty at relaxation, two cluster-like wurtzite AlN and InN crystals were brought together (at initial distance of 2.0 Å along the direction of the *x* axis) and relaxed, Figure 2 b**.** The sizes of the cluster-like wurtzite AlN and InN crystals were then gradually increased from 64, to 128, to 192, and, at the end, to 256 atoms each. The bonding in the interface layer was relaxed in each case, while the remaining parts of the model system were kept frozen, as in previously relaxed wurtzite AlN and InN bulk and slab models. The thickness of the interface layer allowed to relax has been varied (up to 3 nearest neighbor atoms at each side of the interface, thus iteratively improving the approximation that results from employing a large but still finite model system sections and freezing the bonding of parts of them) until the variation in *E*_coh/at_ remains below 0.02 eV/at. This is the most accurate value that we were able to achieve by employing the iterative method.

*E*_bl_ is trivially obtained by multiplying the value of E_coh/at_ of the optimized interfacial system by its corresponding number of atoms. This procedure ensures a computationally efficient estimation of the *E*_bl_ with good accuracy since it is matched to a DFT relaxation at PBC for any comparable model system consisting of group III nitrides. Determination of *E*_bl_, and its substitution in eq 1, together with that of the other (more trivially calculated) energy values, permits computation of the interfacial energy γ_i_^AlN-InN^. The description of the calculation is given for the interfacial energy γ_i_^AlN-InN^ in the benchmark case of interface between the pure binaries AlN-InN which results in the value of 979.2 mJ m^–2^, but the computation of the interfacial energy corresponding to the interfaces between two InAlN phases [(1) and (2), Figure 2 and Table 1] in self-induced core–shell NRs follows the same equation and algorithm.

Once this procedure for calculation of the interfacial energy for interfaces consisting of wurtzite binary compounds is established and judged reliable enough, it can be employed for the determination of the interface energies between ternary InAlN compounds that more closely reproduce the composition of typical cores and shells of experimentally verified self-induced NRs.^18^ In Table 1, the interfacial energies for typical interfaces that are particularly relevant to the experimental results are reported, namely, (i) Al(0.75)In(0.25)N (indium rich core) versus Al(0.98)In(0.02)N (indium poor shell)^7^ as well as three cases of other compositions: of relevance (ii) Al(0.70)In(0.30)N versus Al(0.99)In(0.01)N; (iii) Al(0.79)In(0.21)N versus Al(0.89)In(0.11)N and (iv) Al(0.89)In(0.11)N versus Al(0.97)In(0.03)N. The interfaces (ii) to (iv) are of relevance to core–shell InAlN NRs with broader compositional range and they have been taken into consideration for comparative and testing purposes of PFM simulation runs (which are described in Sec. 3.3 below).

The values of the interfacial energies (Table 1) depend nonlinearly on phase compositions and vary significantly as a function of the core/shell compositions. Qualitative analysis indicates that lower interfacial energy values tend to be obtained for interfaces which have anyway been confirmed experimentally by the growth of the self-induced core–shell InAlN NRs [e.g., Al(0.75)In(0.25)N to Al(0.98)In(0.02)N], or are similar to such and thus experimentally obtainable, although possibly not yet synthesized [e.g., Al_0.89_In_0.11_N to Al_0.97_In_0.03_N]. Such values point out thermodynamic advantages during growth for NRs with interfaces like the above-mentioned ones. Contrarily, the benchmark case of an interface between the two pure binaries AlN and InN is revealed as energetically (more) unfavorable. It is inferred that changing synthesis conditions, such as the deposition temperature, may mitigate these energetic advantages and make possible the synthesis of NRs with interfaces significantly different from the energetically favored ones as per Table 1. However, our goal in the present work is not to explore the attainable self-induce InAlN NRs as per the broad spectrum of possible growth conditions, but to demonstrate the capabilities of the DFT-fed PFM to reproduce typical features of experimentally obtained NRs. Further detailed analysis and corresponding illustrations of PFM simulation results on the NRs refer to the lower interfacial energy values listed in Table 1, exemplified by the typical value of 655.3 mJ m^–2^ (which is relevant to NRs with core/shell compositions of Al_0.75_In_0.25_N/Al_0.98_In_0.02_N, respectively).

### 3.2 Diffusion Coefficients

The significant difference in the composition of the core and shell of the NRs manifests itself in concentration gradients of In and Al. As it is well-known, at the interface between two material subsystems of positive mixing enthalpies, these concentration gradients (spontaneously or intentionally formed during growth) drive the diffusion processes across the interface thus lowering the gradients.^38^ In energy terms, the diffusion thus tends to decrease the difference in the chemical potential and so increasing the gradient for negative enthalpy of mixing (Δ*H*_mix_) (i.e., favoring the exothermicity of mixing), thus bringing the whole system closer to thermodynamic equilibrium.

Whether any measurable diffusion happens during the NRs growth process (or in the grown NRs) is governed by the values of the diffusion coefficients for each of the constituent chemical elements. Yet another reason for computing the diffusion coefficients could be to assess whether any measurable change, composition evolution, or interface dilution, would happen due to diffusion over any meaningful time intervals after the NR growth is concluded. Knowledge of accurate values of the diffusion coefficients would permit testing the long-term stability of the NRs, notwithstanding the experimental evidence which so far indicates that the “as grown” self-induced core–shell InAlN NRs kept at room temperature conditions preserve their sharply defined core–shell interfaces over very long-time span.^17,18^ In any case, the main motivation behind our computation of the diffusion coefficients relevant to the self-induced core–shell InAlN is that they are indispensable parameters of the PFM.^5^

The diffusion coefficient for the atom “*x*” in the system “*y*” is well approximated by the Arrhenius equation for the temperature dependence of reaction rates and thus is defined as
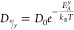
10where *D*_0_ is a pre-exponential factor, which reads *D*_0_ = α*g*υ*L*^2^, i.e., it depends on the parameters α and *g*, which characterize the system where the diffusion takes place, while υ is the hopping frequency, and *L* is the hopping distance between the newly formed vacancy site and the site of diffusion. The hopping distance *L* varying in the range of ∼2–6 Å is extracted from the DFT simulations of generic and specific group III nitride model systems. While the parameters α and *g* may depend on structural anisotropy, for the purposes of the PFM and accounting for mesoscopic scale and the clearly defined core/shell morphology of the NRs, α = *g* can be confidently set as equal to 1.^39^ For solid-state semiconductor binaries and for the temperatures ranges of interest for the NRs growth (∼400–800 °C), the hopping frequency υ is usually set to 1 THz.^39^ Strictly speaking, the pre-exponential factors α = *g* and the hopping frequency υ are the only parameters employed in the hereby proposed DFT-fed phase field model for semiconductor nanostructures that are not derived from DFT calculations carried out in this work but instead estimated from the literature for the type of NRs compounds and their conditions of growth. *E*_A_^y^ is the activation energy for vacancy creation and hopping initiation (in material system with defined composition and structure designated “*y*”), while *k*_B_ and *T* are the Boltzmann constant and the absolute temperature.^40^ For the calculation of the diffusion coefficients for the purposes of this work was adopted the temperature of 700 °C (973 K), a typical temperature for the growth of self-induced core–shell InAlN nanorods.^18^

The role of the DFT calculations in determining the diffusion coefficients (eq 10) consists in evaluating the activation energy *E*_A_^y^ for the Al, In, and N atoms in the context of different interfaces, e.g., Al(0.75)In(0.25)N (core) vs Al(0.98)In(0.02)N (shell).

**Figure 3:**
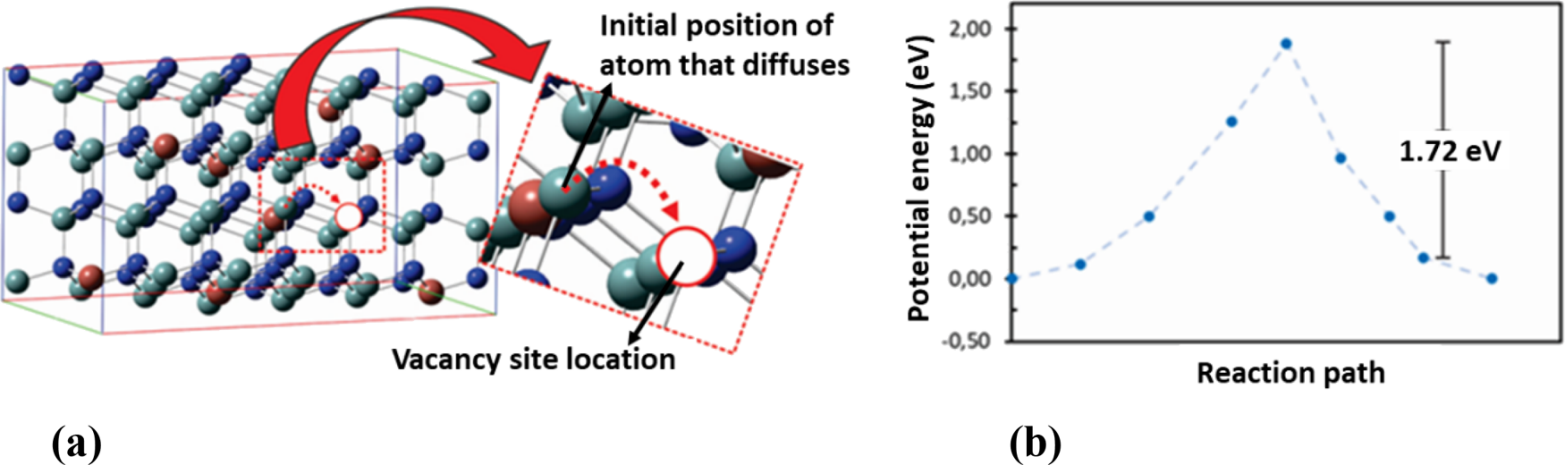
Determination of the diffusion coefficients for N, In, and Al atoms for variety of compositions representing typical cores and shells of the InAlN NRs. (a) Illustration of a vacancy creation and a hopping event exemplified by an Al atom diffusion in wurtzite Al(0.75)In(0.25)N; (b) example of determination of the activation energy by energy barrier calculation employing the nudged elastic band (NEB) in the case of diffusion of an Al atom in NRs with a composition Al(0.75)In(0.25)N corresponding to a reaction path of Al atom diffusing from a shell InAlN crystal site to a vacancy site.

The diffusion coefficients were computed for a range of core/shell compositions of experimental relevance.^18^ A model system illustrating the computation of the diffusion coefficients is shown in Figure 3 a.

The activation energies *E*_A_^y^ in all cases were obtained by computation of the energy barriers to the hopping/diffusion event seen as a chemical reaction and realized by employing the nudged elastic band (NEB) method, Figure 3 b as usually done for the task of determination of the diffusion coefficients, and according to the DFT calculation details provided in Sec. 2.1.

The diffusion coefficients for N, Al, and In atoms and for a variety of NR core–shell compositions are listed in Table 2. The diffusion coefficients for the Al, In, and N atoms in the InAlN compounds relevant to the NRs exhibit values of varying magnitudes, which for the Al and In atoms generally fall in the range 10^–13^–10^–14^. On the other hand, the N atoms diffuse much quicker as indicated by diffusion coefficients exceeding those of the metal atoms by about 6 orders of magnitude. The results in Table 2 illustrate a trend of the Al/In diffusion coefficients versus composition of the studied compound. Namely, for In-richer compositions, e.g., Al(0.75)In(0.25)N, as in a NR core, the diffusion coefficients for Al and In differ less (*D*_Al_/*D*_In_ < 10), while for In-poorer compositions, as in a NR shell, the Al/In diffusion coefficient ratio is more pronounced.

**Table 2:** Diffusion Coefficients for a Variety of Compositions Representing Typical Cores and Shells of the InAlN NRs^a^

diffusion coef. m^2^s^–1^InAlN compounds	Al atoms	In atoms	N atoms
Al(0.75)In(0.25)N	3.91 × 10^–13^	6.54 × 10^–14^	4.63 × 10^–7^
Al(0.79)In(0.21)N	3.43 × 10^–13^	5.82 × 10^–14^	4.87 × 10^–7^
Al(0.89)In(0.11)N	2.46 × 10^–13^	3.94 × 10^–14^	5.22 × 10^–7^
Al(0.97)In(0.03)N	1.61 × 10^–12^	2.20 × 10^–14^	5.49 × 10^–7^
Al(0.98)In(0.02)N	1.30 × 10^–12^	1.80 × 10^–14^	5.66 × 10^–7^

a All diffusion coefficients were calculated for the temperature of 700 °C (973 K), which is considered an optimal temperature for the growth of self-induced core-shell InAlN nanorods.^18^

To our knowledge, there are presently no directly comparable results for estimation of the diffusion coefficients of Al, In, and N in the context of the ternary nitride compounds grown by MSE. However, the importance of accurate determination of the diffusion coefficients values for understanding the formation of group IIIA nitride compounds and nanostructures is well emphasized both theoretically^41^ and experimentally.^42^ The diffusion coefficients calculated in this work for the MSE-grown self-induced core–shell InAlN NRs (Table 2) fall in the span of the diffusion coefficient values over multiple orders of magnitude relevant to IIIA nitrides recognized in the literature for group IIIA nitride compounds and attributed to the wide diversity of growth methods (MOCVD, MBE, MSE) and corresponding growth conditions.^42^

In the markedly disequilibrium conditions characterizing PVD processes (including MSE), the external deposition flux competes with bulk diffusion fluxes of In and Al, which entirely destabilizes any equilibrium or balance between the net atomic flux of In and Al and the vacancy flux. Consequently, modeling the system in terms of the atomic diffusion of the three (in the case of the InAlN NRs) atomic species efficiently covers for the deposition process without explicit consideration of purely vacancy diffusion.

### 3.3 Growth Simulations of InAlN NRs by PFM

While the interfacial energies and the ratios of atomic diffusion constants selected for phase field simulations are based on DFT simulations, the remaining parameters tabulated in Table 3 are chosen based on our prior knowledge on phase separation in ternary alloy films nanostructures.^7^ It is to be noted in such systems that, during the sputtering of phase-separating ternary alloy films, the resulting nanostructures can vary significantly depending on factors such as composition, deposition rate, and temperature (which influence atomic mobility). These variations can lead to distinct nanostructured variants, including those with lateral concentration modulations (LCM), vertical concentration modulations (VCM), and structures with random bicontinuous, core–shell, or globular morphologies. These findings were summarized using morphology maps, as shown in our previous work.^7^ Hereby, we focus on the nanorod morphology, which in 2D looks similar to LCM. To determine the appropriate PFM simulation parameters (Table 3), we ran tests simulating the InAlN NR structures, and we utilized our knowledge of the parameter combinations that result in LCMs from our previous research.^7^ This allowed us to narrow down the parameter space for which NRs of InAlN with a core–shell morphology are expected to form.

**Table 3:** List of PFM Parameters Employed in the Present Work

parameter	value (nondimensional)
Δ*x*	1.0
Δ*t*	0.00005
*T*	1.0
χ_Al, In_	4.0
χ_In, N_	4.2
χ_Al, N_	4.2
κ_*ii*_ (*i* = Al, In, N)	7.0
κ_*ij*_ (*i* = Al, In, N)	4.0
*A*	0.001
*D*_Al_	1.0 (core), 0.3 (shell)
*D*_In_	0.15 (core), 0.05 (shell)
*D*_Al_^*S*^	3.0
*D*_In_^*S*^	0.45
*L*	0.01
*n*	15

**Figure 4:**
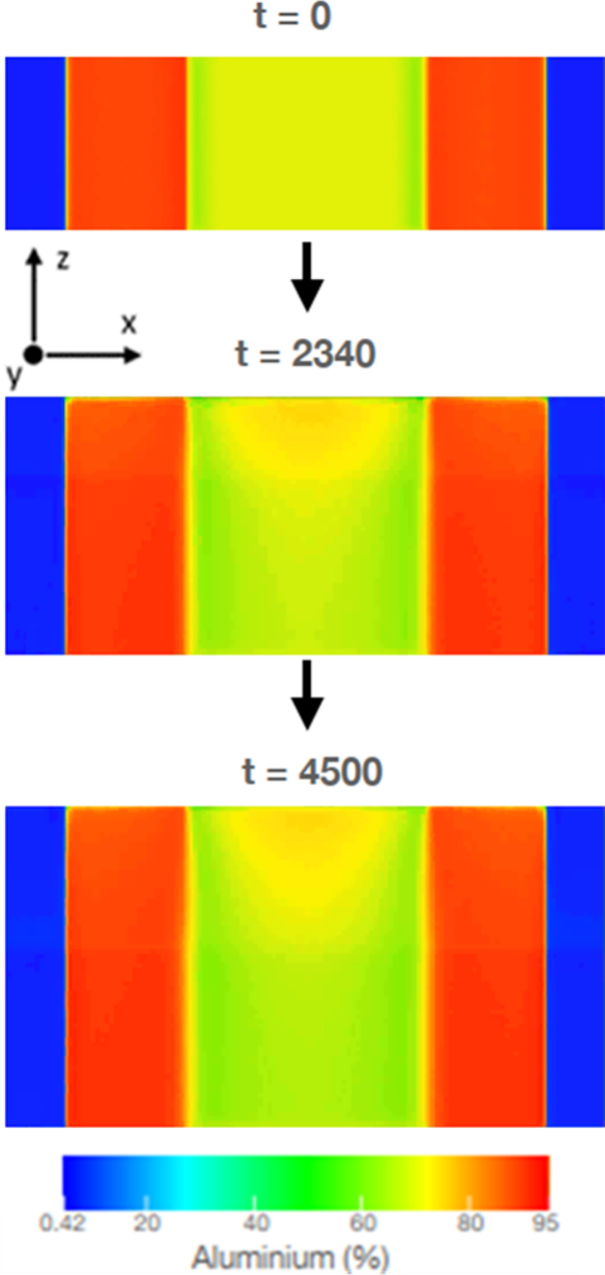
Representative snapshots showing the two-dimensional evolution of a single NR that are simulated using PFM. Colors correspond to the concentration of Al (see legend at the bottom of the figure). Thickness is measured along the deposition axis (*z* axis), which is denoted as the *z* axis. In the 2D simulations adopted in this work, this is the same as the shorter edge of the seed shown in the left panel (*t* = 0).

PFM simulations of InAlN NR growth were performed using a discretized deposition flux that incorporates at least one layer of noisy unseparated layer on the film’s surface. This technique is very similar to the one adopted in our previously reported model that is able to replicate realistic nanostructures in immiscible alloy films.^4−6^ A two-dimensional simulation of NR evolution starting from the nanostructured seed is shown in Figure 4.

**Figure 5:**
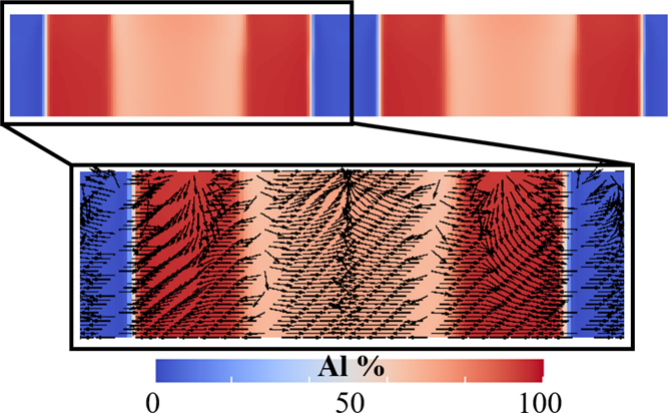
Evolution of the Al composition map simulated at the nondimensional time, *t* = 2340. The tiny black arrows illustrate the Al diffusional fluxes that are superimposed on the composition map. The indicated composition is in % of total quantity of metal atoms (Al + In correspond to 100% metal atoms). Only the metal composition is visualized in the color-coded map. The N concentration (not visualized) is assumed as 50% of all NRs atoms since InN and AlN are essentially stoichiometric compounds.

The simulated Al diffusional fluxes within the film, which are calculated by computing compositional gradients, are plotted over the Al concentration map in Figure 5. Here, the PFM simulations indicate that the Al- and In-rich phases grow in a columnar fashion, as the phase interface remains perfectly aligned along the surface normal during the deposition. In other words, the morphology of phases in the seed remains preserved during growth due to the perfect redistribution of Al and In from the unseparated layer into both the growing phases. However, the Al concentration is found to increase toward the center of the In-rich region. This can be attributed to the fast diffusion of Al into the In-deficit or Al-rich phase from the AlN/InN phase boundary, as visualized by the direction of diffusional fluxes plotted in Figure 5.

**Figure 6:**
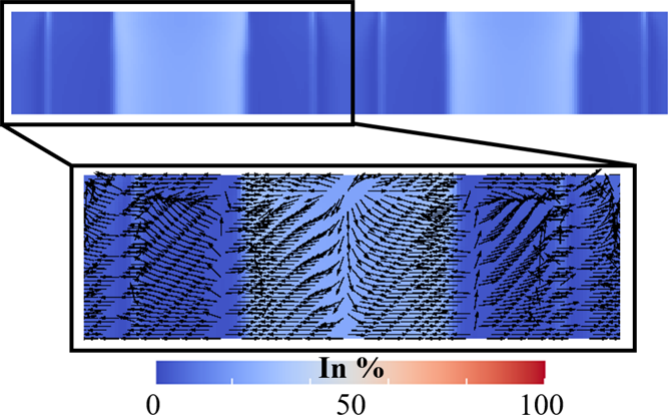
Evolution of the In composition map simulated at the nondimensional time, *t* = 2340. The tiny black arrows illustrate the In diffusional fluxes that are superimposed on the composition map. The indicated composition is In % of total quantity of metal atoms (Al + In correspond to 100% metal atoms). Only the metal composition is visualized in the color-coded map. The N concentration (not visualized) is assumed as 50% of all NRs atoms.

Here, the red shade corresponds to pure Al, whereas the blue represents the vapor phase, which is predominantly composed of N. The color grading within the red indicates the redistribution of Al in Al-rich and In-rich phases, respectively. On the contrary, diffusional In flux is found to be directed toward the In-rich phase, as seen in Figure 6. The diffusional fluxes of Al atoms (Figure 5) and In atoms (Figure 6) reveal the diffusion of In atoms from the In-poor NR shell to the In-rich NR core and the diffusion of Al atoms from the Al-poorer NR core to the Al-richer NR shell. This PFM result explicates the driving force behind the experimentally observed composition of the InAlN NRs as directly resulting from the characteristics–the interfacial energies and the diffraction coefficients–inherent to the ternary InAlN compounds from which the NRs are formed.

The diffusional fluxes depicted in Figures 5 and 6 are illustrative for how and why the immiscibility gap, ranging 0.1 < *x* < 0.9 for InAlN bulk phases,^43,44^ is being reduced to (at least) 0.25 < *x* < 0.75 in InAlN nanostructures whose formation is ruled at nanoscale size ranges. This immiscibility gap mitigation observed here in 2D PFM simulation is a phenomenon inherent to the core–shell InAlN NRs, which is corroborated by detailed 3D DFT-based synthetic growth concept simulations of the InAlN NRs (albeit in a size scale much smaller than PFM) in our previous work accounting for prevalence-related characteristics of the precursors’ species and their energetics during the NR growth.^24^ The occurrence of such a phenomenon has also been suggested for other material systems in nanoscale, e.g., AuCo nanoalloys, which similar to InAlN exhibit immiscibility as bulk phases.^45^

**Figure 7:**
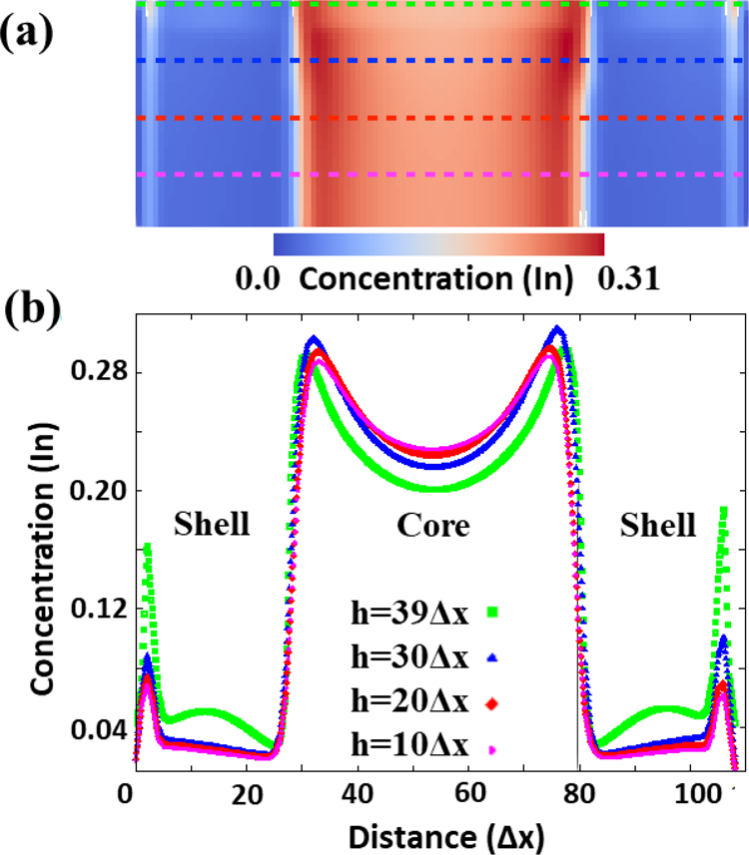
(a) Simulated In concentration map at *t* = 3540. (b) 1D In concentration profiles plotted at different heights ‘*h*’. Plot color corresponds to the lines plotted in panel (a).

At this point, it is worth analyzing In diffusion within the evolving nanorod to deduce mechanistic pathways of phase separation. Figure 7 shows the In concentration line plots across a simulated nanostructure at different thicknesses. Closer to the top surface, a downward gradient within the Al-rich shell redistributes In atoms toward the core–shell interface and the nanorod lateral surface. However, descending down the rod thickness, a sustained In gradient pointing toward the core–shell interface develops. The plots in Figure 7 clearly indicate that the nature of the diffusional transport within the nanorod favors the growth of the In-rich core.

It is worth noting that the PFM simulation leading to this result employs as parameters only the InAlN NR properties and characteristics (interfacial energies and diffusion coefficients), which are obtained via ab initio procedures of high accuracy (Sec. 3.1 and 3.2). No phenomenological or any other experimentally derived parameters have been used in the PFM simulation runs, which makes the hereby reported PFM results ab initio-based implying the corresponding accuracy. It is also an advantage that the PFM simulations and results are not dependent upon any present or future availability of experimentally derived or phenomenological parameters and their possible inaccuracies but are entirely computationally self-consistent.

Another essential experimentally observed feature of the InAlN nanorods, as revealed by the PFM simulations, concerns the confirmed stable and planar (cylindrical in 3D) core–shell interface. Thus, during the PFM simulation runs, the NR core/shell phase separation emerges naturally, while during the subsequent PFM growth, this interface is kept spontaneously planar. Notably, this most characteristic self-organization feature of the InAlN NRs appears naturally emerging, and then, it is consistently preserved during the PFM simulation.

As explained in Sec 2.2 as well as directly above in relation to the choice of the parameters of the PFM simulations, the nondimensional deposition rate is set as relatively slow as based on previous experience in running PFM simulations of phase-separating ternary alloy films^7^ and is thus not directly related to any experimental deposition rate. At a slow nondimensional deposition rate, Al and In atoms are permitted sufficient time to redistribute into the respective Al- and In-rich phases. As a result, the phase interfaces stabilize into a planar morphology, which in 3D would translate to the well-known cylindrical core–shell morphology.^10^

At possibly faster deposition rates, which have not been covered in this work, one could expect nanostructure variants different from the characteristic core–shell NRs to form. Although the scope of the current model is limited to 2D, the simulated nanorod is realistic enough to correspond to the cross-section of the ones synthesized by reactive magnetron sputter epitaxy.^44,46^ A 3D phase-field model, which we intend to develop further, would allow the incorporation of 6-fold symmetry with the possibility of including additional phases within the core–shell nanostructure. Such a model would also be suitable for testing faster deposition rates.

## 4 Conclusions

We incorporate a DFT level of theory in mesoscopic modeling of the NRs through the purposefully developed/adapted phase field model (PFM). Thus, we apply state-of-the-art DFT methodology to develop procedures for computation of the interfacial energies and the diffusion coefficients of Al, In, and N atoms in compounds with compositions experimentally relevant to the self-induced core–shell InAlN NRs (e.g., Al(0.75)In(0.25)N to Al(0.98)In(0.02)N). We also discuss aspects and choices pertinent to the development of the DFT procedures for the calculation of interfacial energies and diffusion coefficients and the fitness/adaptation of these calculation procedures to InAlN NRs.

The morphological evolution of the In- and Al-rich compound regions simulated by PFM in the current work resembles the 2D cross section of InAlN nanorods that have previously been reported in the literature. The PFM simulations confirm and visualize the diffusion of In atoms from In-poor NRs shell to the In-rich NRs core and the diffusion of Al atoms from Al-poorer NR core to the Al-richer NR shell. This essential PFM result accurately reproduces one of the most basic features of InAlN NRs. The interface between the phases is predicted to be planar and stable during the course of deposition since a slow deposition rate allows sufficient time for Al and In to redistribute into their respective phases. In the future, it will be interesting to simulate nanorod growth at faster deposition rates that could potentially destabilize the core–shell morphology.

The presented DFT-PFM simulation approach, as hereby demonstrated in a case study of the self-induced core–shell InAlN NRs, can open the way for PFM applications to a wide range of semiconductor nanostructured materials and low-dimensional systems of complex morphology and composition.
